# Olezarsen: FDA approval and clinical impact in familial chylomicronemia syndrome (FCS)

**DOI:** 10.1097/MS9.0000000000003768

**Published:** 2025-09-04

**Authors:** Muhammad Saad Khan, Devya Khaim Chandani, Erum Siddiqui, Maliha Khalid, Aminath Waafira

**Affiliations:** a Department of Medicine, Jinnah Sindh Medical University, Karachi, Pakistan; b Department of Medicine, Shaheed Mohtarma Benazir Bhutto Medical College, Karachi, Pakistan

**Keywords:** familial chylomicronemia syndrome, hypertriglyceridemia, lipoprotein lipase, olezarsen, pancreatitis

## Abstract

Familial chylomicronemia syndrome (FCS) is a rare autosomal recessive disorder characterized by severe hypertriglyceridemia due to impaired lipoprotein lipase (LPL) function. Traditional treatments like dietary fat restriction and conventional lipid-lowering drugs offer limited benefit due to the underlying genetic deficiency. On December 19, 2024, the Food and Drug Administration approved olezarsen (Tryngolza), an antisense oligonucleotide targeting apolipoprotein C-III (apoC-III), for adults with FCS. By inhibiting apoC-III synthesis, olezarsen enhances LPL activity and facilitates triglyceride clearance. Phase 3 trials demonstrated a significant reduction in triglyceride levels and a marked decrease in pancreatitis episodes, establishing its therapeutic efficacy. Olezarsen is administered monthly via subcutaneous injection, with most adverse events being mild and transient, such as injection site reactions and occasional thrombocytopenia. While short-term outcomes are promising, long-term safety, cost-effectiveness, and broader accessibility remain key concerns. Furthermore, the drug exemplifies the integration of computational biology and precision medicine, laying the foundation for AI-driven innovations in managing rare lipid disorders. Overall, olezarsen represents a major advancement in FCS treatment, addressing an urgent unmet clinical need and reshaping the therapeutic landscape of ultra-rare metabolic diseases.

On December 19, 2024, the U.S. Food and Drug Administration (FDA) approved Tryngolza (olezarsen) as an adjunct to diet for lowering triglycerides (TGs) in adults with familial chylomicronemia syndrome (FCS)^[1]^. While this marks a first-in-class approval, signifying a novel mechanism of action, it’s crucial to understand its place among existing FCS management strategies. Current approaches, such as strict dietary fat restriction and supportive care often fail to adequately control TG levels and prevent complications like pancreatitis. Unlike traditional lipid-lowering therapies like statins and fibrates, which are ineffective in FCS due to the underlying genetic defects affecting lipoprotein lipase function, Olezarsen directly addresses the overproduction of apoC-III, a key regulator of TG metabolism. This targeted approach offers a new avenue for managing FCS, particularly for patients who struggle to achieve adequate TG control with diet alone and remain at high risk for pancreatitis.

## Introduction

Familial chylomicronemia syndrome (FCS) is a rare genetic disorder associated with impaired LPL function, resulting in severe hypertriglyceridemia and recurrent acute pancreatitis. It typically manifests in the first few years of life with eruptive xanthomas, hepatosplenomegaly, lipemia retinalis, and recurrent acute pancreatitis, although some cases go undiagnosed until adulthood^[2]^. Neurological symptoms such as cognitive impairment, fatigue, memory problems, and mood disorders such as depression and irritability are also reported, presumably a consequence of impairment of lipid metabolism within the brain. Common gastrointestinal symptoms include nausea, vomiting, diarrhea, and bloating, which tend to be aggravated by high-fat meals^[3]^. This manuscript has been developed in accordance with the TITAN Guidelines (2025) (Table 1)^[4]^.

**Table 1:** TITAN Guideline Checklist 2025

Topic	Item	Description	Page number
Artificial intelligence (AI) (some journals may prefer this in the methods and/or acknowledgments section and, it should also be declared in the cover letter)	1	Declaration of whether any AI was used in the research and manuscript development	(NO)
		State no, if that’s the case.	
		If yes, proceed to item 1a.	
	1a	Purpose and scope of AI use Precisely state why AI was employed (e.g. development of research questions, language drafting, statistical analysis/summarization, image annotation, etc). Was generative AI utilized and if so, how? Clarify the stage(s) of the reporting workflow affected (planning, writing, revisions, figure creation). Confirmation that the author(s) take responsibility for the integrity of the content affected/generated	
	1b	AI tool(s) and configuration Name each system (vendor, model, major version/date). State the date it was used Specify relevant parameters (e.g. prompt length, plug-ins, fine-tuning, and temperature). Declare whether the tool operated locally on-premises, or via a cloud API and any integrations with other systems.	
	1c	Data inputs and safeguards Describe categories of data provided to the AI (patient text, de-identified images, literature abstracts). Confirm that all inputs were de-identified and compliant with GDPR/HIPAA. Note any institutional approvals or data-sharing agreements obtained.	
	1d	Human oversight and verification Identify the supervising author(s) who reviewed every AI output. Detail the process for fact-checking and clinical accuracy checks State whether any AI-generated text/figures were edited or discarded. Acknowledge the limitations of AI and its use	
	1e	Bias, ethics, and regulatory compliance Outline steps taken to detect and mitigate algorithmic bias (e.g. cross-checking against underrepresented populations). Affirm adherence to relevant ethical frameworks. Disclose any conflicts of interest or financial ties to AI vendors.	
	1f	Reproducibility and transparency Provide the exact prompts or code snippets (as supplementary material if lengthy). Supply version-controlled logs or model cards where possible. if applicable, state repository, hyperlink, or digital object identifier (DOI) where AI-generated artefacts can be accessed, enabling attempts at independent replication of the query/input.	

## Epidemiology

The estimated global prevalence of FCS is approximately 1 in 100 000–1 000 000 individuals^[5]^. Recent research suggests that the prevalence of FCS may be higher than previously estimated due to underdiagnosis and misclassification of multifactorial hypertriglyceridemia^[6]^. The carrier frequency of LPL gene mutations is about 1 in 500, indicating that heterozygous individuals are reasonably prevalent yet do not get the complete syndrome. Men and women are affected equally, but females may have worsening TG levels during pregnancy or when on oral contraceptives, thought to be secondary to the effects of hormones. Specific populations, including French Canadians, the Dutch, and some Asian groups, have higher rates because of founder effects and consanguinity^[7]^.

## Pathophysiology

LPL enzyme is essential for hydrolyzing TG-rich lipoproteins, mainly chylomicrons and very low-density lipoproteins (VLDL)^[8]^. The primary genetic defect in FCS is in the LPL gene, which encodes the lipoprotein lipase enzyme responsible for hydrolyzing TGs^[4]^. Mutations in the LPL gene cause either the entire absence or severe dysfunction of LPL, impeding the breakdown of circulating TGs^[8]^. This leads to hypertriglyceridemia of the severe type, with TG levels frequently greater than 1000–2000 mg/dl, which in turn increases blood viscosity and risk of pancreatitis^[8,9]^. Contributing factors such as alcohol, estrogen therapy, and pregnancy can exacerbate hypertriglyceridemia.

## Diagnosis, current management, and therapeutic gaps

Laboratory findings include severe hypertriglyceridemia, sometimes >1000 mg/dl, the milky appearance of fresh plasma, low apolipoprotein B levels, and low levels of post-heparin-plasma LPL activity. However, genetic testing is a gold standard that confirms the diagnosis by identifying homozygous or compound heterozygous mutations in genes involved in TG metabolism^[2]^. Management of FCS involves a multipronged approach to reduce chylomicron levels and mitigate the risk of pancreatitis. Conventional lipid-lowering treatments, such as statins, fibrates, and omega-3 fatty acids, are mainly ineffective in FCS due to a genetic deficiency in LPL function. Fibrates activate PPAR-α to enhance LPL expression, while omega-3 fatty acids inhibit hepatic VLDL synthesis. However, these strategies are not effective if LPL is either absent or non-functional^[10]^. Studies have shown that these medicines had limited to no TG decrease in FCS patients, rendering them inappropriate as stand-alone therapies^[11]^.

Strict dietary fat restriction (typically <10–20 g/day) is the cornerstone of management for FCS, as it does not respond well to conventional TG-lowering treatments^[12,13]^. However, maintaining such a regimen over time is quite difficult. Adherence is frequently jeopardized due to limited food options, social constraints, and a significant impact on quality of life. Furthermore, very low-fat diets raise the risk of fat-soluble vitamin deficiencies (A, D, E, and K) and essential fatty acid deficiency, which may necessitate ongoing supplementation and monitoring^[12,14]^.

Other supportive therapies have been used to alleviate symptoms and improve nutritional intake. Medium-chain triglyceride oil supplements are commonly used because they are absorbed directly through the portal vein and do not require chylomicron formation, preventing postprandial TG surges^[15]^. Pancreatic enzyme replacement therapy may be used to aid in nutrient absorption in patients with recurrent pancreatitis or concomitant pancreatic insufficiency^[16]^.

Despite these therapeutic interventions, many FCS patients continue to have severely increased TG levels, recurring acute pancreatitis, gastrointestinal discomfort, and psychosocial stress. This demonstrates a clear unmet need for targeted pharmacologic treatments that address the underlying pathophysiology while maintaining metabolic control.

## Olezarsen: mechanism of action

**Figure 1.**
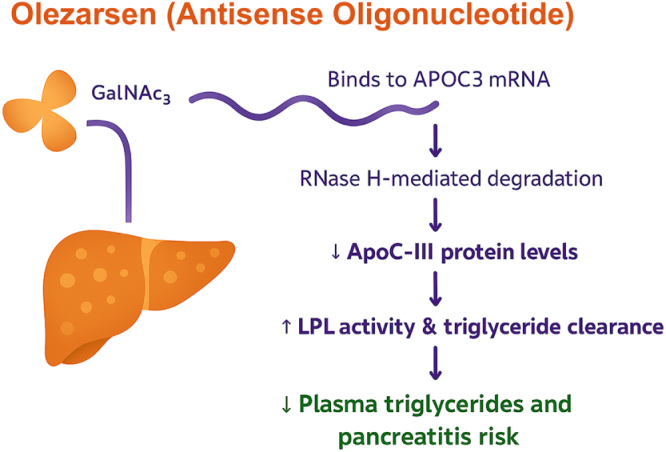
Mechanism of action of Olezarsen (Antisense Oligonucleotide)

Olezarsen is an antisense oligonucleotide designed to inhibit the synthesis of apolipoprotein C-III (apoC-III), a key inhibitor of LPL activity. By binding to apoC-III mRNA and promoting its degradation through RNase H-mediated cleavage, it reduces apoC-III protein levels, enhances LPL activity, and facilitates TG clearance^[17]^ (Fig. 1).

It is given as a subcutaneous injection (usually 80 mg monthly) and conjugated with a triantennary N-acetylgalactosamine (GalNAc3) ligand. This GalNAc3 moiety allows for high-affinity, receptor-mediated transport to hepatocytes via the asialoglycoprotein receptor, which is widely expressed in the liver. This site-specific targeting enhances drug uptake by the liver, the central site of apoC-III synthesis, while reducing systemic exposure^[18,19]^.

The GalNAc3 conjugation strategy improves drug specificity and potency, enabling lower and less frequent dosing. It also reduces systemic side effects and enhances patient compliance by allowing monthly administration. Once inside hepatocytes, olezarsen is metabolized via endonuclease cleavage into shorter fragments and subsequently degraded to nucleotides by exonucleases^[20]^. It has an approximate terminal half-life of 4 weeks.

## Summary of clinical trials

A recent Phase 3 randomized controlled trial (RCT) evaluated the safety and efficacy of olezarsen in 66 patients with genetically confirmed FCS. Participants were randomized in a double-blind manner to receive either 50 or 80 mg of olezarsen subcutaneously every 4 weeks, or placebo, over a 49-week period. Eligible patients had persistent severe hypertriglyceridemia and a documented history of pancreatitis. The primary endpoint was the percent change in fasting TG levels from baseline^[21]^

The 80 mg group achieved a 43.5 percentage point reduction in TGs compared to placebo (*P* < 0.001), whereas the 50 mg group did not reach statistical significance. Notably, during the 53-week follow-up, only one acute pancreatitis episode was reported in each olezarsen group versus 11 episodes in the placebo group, highlighting the drug’s efficacy in reducing pancreatitis risk^[21]^ (Table 2).

**Table 2:** Summary of clinical trials

Trial name	Population	Dose	TG reduction (%)	Pancreatitis events	Other outcomes
Phase 3 RCT	66 FCS patients	80 mg SC every 4 weeks	43.5% (placebo-corrected)	2 vs 11 (placebo)	Significant ApoC-III reduction
BALANCE Trial	66 FCS patients	50 and 80 mg SC monthly	59.4% (12 mo, 80 mg)	2 vs 11 (placebo)	ApoC-III ↓ up to 81.3%
RCT (50/80 mg)	154 Hypertriglyceridemia patients	50 mg/80 mg monthly	49.3%/53.1%	Not reported	↓ ApoB, non-HDL-C
Phase 1/2a Study	Dose-escalation in hypertriglyceridemia	10–120 mg (single dose)	−12% to −77%	Not reported	Dose-response relationship seen

Secondary endpoints included reductions in apoC-III (up to 81.3%), apoB, and non-HDL cholesterol. However, no significant changes were observed in patient-reported quality of life. Limitations of the study include the small sample size, relatively short duration given the chronic nature of FCS, and exclusion of patients with advanced hepatic or renal dysfunction. Adverse events such as injection site reactions, arthralgia, and transient thrombocytopenia were observed, underscoring the need for continued post-marketing surveillance^[21]^.

The Phase 3 BALANCE study evaluated the efficacy and safety of olezarsen in 66 patients with genetically confirmed FCS. The average baseline TG level was ~2600 mg/dl, and 71% (47/66) of patients had a history of acute pancreatitis. This double-blind, placebo-controlled trial assigned participants to receive either 50 or 80 mg olezarsen subcutaneously every 4 weeks. At 6 months, the placebo-adjusted TG reductions were -43.5% (*P* = 0.0009) in the 80 mg group and -22.4% (*P* = 0.0775) in the 50 mg group. These reductions deepened at 12 months to -59.4% and -43.8%, respectively^[22]^.

Notably, apoC-III levels dropped significantly at both timepoints: -65.5% and -73.7% at 6 months and -77.1% and -81.3% at 12 months for the 50 and 80 mg groups, respectively. Secondary endpoints revealed substantial reductions in apoB and non-HDL cholesterol. Acute pancreatitis episodes occurred in only two patients receiving olezarsen (one per treatment group), compared to 11 episodes in the placebo group, reinforcing olezarsen’s role in mitigating life-threatening complications^[22]^ (Table 2).

However, limitations included the relatively small sample size, short trial duration for a chronic disease, and the exclusion of patients with hepatic or renal comorbidities. While adverse events were mostly mild such as injection site reactions, arthralgia, and transient thrombocytopenia long-term surveillance remains essential^[22]^

Another RCT reported monthly olezarsen (50 or 80 mg) reduced TG levels by 49.3% and 53.1%, respectively (*P* < 0.001 for both), with significant decreases in apolipoprotein C-III, apolipoprotein B, and non-HDL cholesterol, and no major safety concerns, confirming its efficacy in lipid management^[23]^.

A phase 1/2a, double-blind, placebo-controlled dose-escalation research in individuals with hypertriglyceridemia found that a single dose of olezarsen 10, 30, 60, 90, or 120 mg resulted in a median reduction in plasma TG levels of −12%, −7%, −42%, −73%, and −77% after 14 days^[24]^.

## Safety profile and adverse events

Olezarsen was generally well-tolerated in clinical trials. However, mild to moderate adverse effects were common and warrant clinical consideration. Injection site reactions such as erythema, pain, or swelling – were the most frequently observed events, occurring in approximately 18% of participants receiving the 80 mg dose and were typically self-limiting. Arthralgia was also reported, affecting 2–3 patients per group, but rarely led to treatment discontinuation^[25]^.

Of particular note, transient thrombocytopenia (defined as platelet count <140 000/mm^3^) occurred in 9% of the 80 mg group, 19% in the 50 mg group, and 17% of placebo recipients, with no associated bleeding events^[21,23]^. These declines in platelet count were reversible and did not necessitate permanent cessation of therapy. Hypersensitivity reactions were rare but potentially serious, underscoring the importance of monitoring for signs of anaphylaxis. As a precaution, olezarsen is contraindicated in patients with known severe hypersensitivity to the drug or its components.

While short-term safety outcomes were favorable, the study duration was limited to 53 weeks. Therefore, long-term surveillance is essential to assess cumulative risks, particularly with chronic use. Continued pharmacovigilance is also warranted to monitor for rare but serious immune-mediated or hepatic complications.

## Future prospects

Future directions for olezarsen involve not only expanding its therapeutic applications but also establishing its role in the long-term management of FCS, a chronic, lifelong metabolic disorder. While initial clinical trials have shown promising reductions in TG levels and pancreatitis incidence, these results are based primarily on short- to mid-term data (6–12 months), which are insufficient for evaluating long-term durability and safety.

To address this gap, ongoing and future studies must prioritize the collection of long-term efficacy and safety data, including post-marketing surveillance and real-world evidence across diverse patient populations. This includes patients with hepatic or renal impairment and those with comorbid conditions, who may have distinct risk profiles or therapeutic responses. Identifying and managing delayed or cumulative adverse events such as immune-mediated reactions, hepatotoxicity, or long-term thrombocytopenia will be crucial for ensuring the safety of chronic use.

Additionally, access and affordability remain significant challenges. As a high-cost biologic therapy, olezarsen poses barriers to equitable distribution, particularly in resource-limited settings. Cost-effectiveness analyses, public health strategies, and innovative pricing models will be vital to ensure broader accessibility.

Collectively, these ongoing efforts will determine olezarsen’s true impact – not only in managing FCS but also in shaping the future of precision lipid-lowering therapies in rare and complex metabolic diseases.

## AI-based perspectives

Olezarsen, the first FDA-approved antisense oligonucleotide for FCS, represents a paradigm shift in lipid disorder management by targeting apoC-III mRNA and regulating TG metabolism at the genetic level^[24]^. This approach represents a new “biological molecular algorithm”: a precision intervention strategy developed through computational analysis of pathogenic gene networks. The drug’s development underscores the increasing confluence of biotechnology and computational biology, providing the groundwork for the integration of AI-powered precision medicine^[25]^.

The introduction of models such as AlphaFold has transformed molecular structure prediction, accelerating drug design and discovery^[26]^. Similarly, AI methods based on clinical imaging and multi-omic data are currently being used to guide personalized medication in oncology and may be applied to uncommon metabolic diseases^[27]^.

Olezarsen thus not only represents a first-in-class therapeutic milestone but also a benchmark for the integration of smart individualization in drug development. Future research should explore how AI can be systematically embedded into clinical trial design, post-marketing surveillance, and real-world treatment personalization for FCS and similar ultra-rare metabolic disorders.

## Conclusion

The FDA approval of olezarsen represents a significant milestone in the management of FCS. It addresses an urgent unmet need in patients who previously had few effective treatment options. It’s in reducing TG levels and preventing pancreatitis, combined with its targeted delivery mechanism, positions it as a cornerstone in the evolving landscape of lipid-lowering therapies. Continued research must explore long-term safety, cost-effectiveness, and AI-driven personalization to optimize its clinical utility.

## Data Availability

No data were generated for this manuscript.
